# Understanding eating disorder symptoms in same-gender couples: social environmental factors

**DOI:** 10.1186/s40337-023-00732-z

**Published:** 2023-01-11

**Authors:** Diane L. Rosenbaum, Kristin J. August, Meghan M. Gillen, Charlotte H. Markey

**Affiliations:** 1 Abington College, Psychological and Social Sciences, Pennsylvania State University, 1600 Woodland Road, Abington, PA 19001 USA; 2 Rutgers University, Camden, USA

**Keywords:** Eating disorders, Sexual minority individuals, Same-gender relationship, Gay, Lesbian, Sociocultural attitudes, Romantic relationship, Ecological systems theory

## Abstract

**Background:**

Sexual minority individuals are at disproportionately greater risk for eating disorders, yet little is known about the ways in which factors in the social environment relate to eating disorder symptoms in this population. Utilizing an ecological systems theory framework, we investigated the relative relationships of macro level (i.e., internalization of sociocultural attitudes about appearance) and micro level (i.e., quality of romantic relationship) social environment influences.

**Methods:**

Men (*n* = 144) and women (*n* = 144) in committed same-gender relationships were recruited as a dyad and completed study questionnaires, including multiple measures of eating disorder symptoms. Multilevel models controlling for key variables (e.g., body mass index) were used to examine gender differences, main effects, and interactions.

**Results:**

Men reported greater internalization of sociocultural attitudes and uncontrolled eating. Greater internalization of sociocultural attitudes was related to greater eating pathology across all measures. For men, greater relationship quality was related to less uncontrolled eating.

**Conclusions:**

Individuals in same-gender relationships experience macro (e.g., sociocultural) level vulnerability factors in relation to disordered eating; however, internalization of sociocultural attitudes may be greater for men. Support at the microsystem level in the form of a high quality committed romantic relationship appears to be helpful protection against uncontrolled eating for men.

## Plain English summary

Sexual minority individuals are at disproportionately greater risk for eating disorders, yet little is known about the ways in which factors in the social environment relate to eating disorder symptoms in this population. This research investigates sociocultural attitudes and romantic relationship quality as factors in the social environment in relation to eating disorder symptoms for men and women in same-gender relationships. We found differences in the occurrence of eating disorder symptoms, such that sexual minority men had more uncontrolled eating compared to sexual minority women. Across all participants, internalization of sociocultural attitudes was linked to greater disordered eating symptoms. We also investigated a potential buffering role of a romantic partner on eating disorder symptoms, and found that high quality, committed, romantic relationships were beneficially associated with less uncontrolled eating for sexual minority men.

## Introduction

Eating disorders (EDs) are prevalent and deleterious, particularly among sexual minority individuals. Some recent research suggests sexual minority men and women are at greater risk for the development of EDs, with higher rates of ED symptoms compared to heterosexual individuals [1]. Moreover, a nationally representative epidemiological survey of men and women found that sexual minority individuals have rates of current ED diagnoses that are 2–5 times higher than heterosexual individuals, and odds of a lifetime ED diagnosis that are roughly double to triple what is found compared to heterosexual individuals [2]. Despite the higher risk and greater prevalence of EDs among sexual minority individuals, relatively little is known about the factors that may contribute to the presentation of weight and eating concerns within sexual minority communities.

Some have theorized that factors in the social environment may relate to the higher likelihood for EDs among sexual minority individuals. For instance, some attitudes and norms within sexual minority subgroups may present ED risk factors [3, 4]. Research indicates that norms within gay communities may promote greater idealization of athletic and lean builds, putting men within the gay community at greater risk compared to heterosexual men [5, 6]. This corresponds with some data that suggest that sexual minority men may be particularly vulnerable compared to heterosexual men to ED symptoms [1]. Research that has acknowledged the potential for risk due to sexual minority community norms has also suggested that the LGBTQIA+ (Lesbian, gay, bisexual, transgender, queer, intersex, asexual, and other gender and sexual identities) community may be a protective factor against ED symptoms [3, 4]. For instance, compared to heterosexual women, women from lesbian communities have been found to accept a broader ranges of body types [7], suggesting the potential for protection against dietary restriction. Greater sense of belonging within the lesbian community has been shown to be protective against negative effects of body dissatisfaction for lesbian women [8]. Taken together, this research suggests there may be gender differences that affect whether sexual minority community norms serve as risk or protective factors in EDs. However, the existing research on the role of sociocultural factors in EDs for sexual minority individuals is limited. Much of the existing research in this area has focused on comparisons of disordered eating behaviors between sexual minority and heterosexual individuals. Additional work is needed to understand differences within sexual minority communities, and the ways in which social environmental factors may heighten or mitigate risk.

### Theory

One theory that has sought to better understand the effects of different factors in the social environment on human functioning is Bronfenbrenner’s [9] ecological systems theory. This developmental model describes four environments that are nested within one another, namely the microsystem, mesosystem, exosystem, and macrosystem. The microsystem is the most proximal setting for an individual and includes the close relationships and roles (e.g., romantic partner) with which an individual has direct contact. The mesosystem includes the interactive connections between factors within the microsystem. The exosystem exists a step beyond an individual’s typical sphere, but could still affect the individual in more indirect ways. The macrosystem includes the norms, ideologies, patterns, and cultural conventions of a society. Bronfenbrenner has described that macrosystems can be implicitly adopted by the people who are part of a society. More specifically, when defining the parts of his Ecological Systems Theory he wrote “most macrosystems are informal and implicit–carried, often unwittingly in the minds of the society’s members as ideology..”(p. 515) [10]. Elements of this theory have been included in previous models of eating behaviors, in which it is noted that sociocultural and interpersonal factors are major influences on eating [11]. These influences fit within the macrosystem (i.e., overarching norms and factors that shape our cultural existence), and microsystem (i.e., the proximal factors which have a role in our individual existence), respectively. The mesosystem and exosystem pose less direct pathways toward influencing the individual, and as such have not yet been established as strong factors in connection to ED symptoms. As an example, the relationship between one’s partner and family (a mesosystem factor) may be less influential on beliefs about weight and shape than either of these microsystem sources independently. Similarly, a romantic partner’s workplace (an exosystem factor) is likely less influential than their partner (a microsystem factor). Therefore, we chose to focus on the macrosystem factor of sociocultural attitudes about weight and shape and the microsystem factor of romantic relationships in our study based on previous work that has established their importance.

It is possible that messages from the macrosystem may be transmitted directly to individuals as well as filtered through the microsystem to relate to eating behavior among sexual minority individuals. Within the microsystem, in adulthood, romantic partners are likely the key figures through which sociocultural attitudes regarding weight and shape are transmitted. In contrast to research involving youth, in which parents are a key source for attitudes about weight [12], some previous research has not found a relationship between parental messages and body dissatisfaction in adults [13]. The influence of parents may be even weaker for sexual minority adults, considering that some individuals consider close others in the LGBTQIA+ community to be a support system (e.g., “chosen family”) that may be as, or more, important than a family of origin [14]. However, there is work to support to the importance of romantic partners in conveying sociocultural messages about weight and appearance in adulthood, and in sexual minority individuals in particular. Specifically, previous work indicates that gay men prioritize perceived attractiveness and thinness in romantic partners [15], but the same pressure is not known to exist for friendships. Similarly, romantic partners develop a level of closeness that may not replicate in other relationships and as such serve as important influences on beliefs and attitudes. That is, since individuals tend to develop a shared sense of identity in the context of romantic relationships [16], the attitudes held by one partner may become part of the shared couple identity over time. This places romantic partners’ attitudes and behaviors regarding eating and weight in a unique position of importance within microsystem factors. Similarly, individuals value the opinions and attitudes of romantic partners above other community members. For instance, the behavior and perceived approval of romantic partners has been shown to predict health behaviors (i.e., smoking) beyond that of friends [17]. Individuals may be influenced, positively or negatively, based on the degree to which their partners adhere to and communicate sociocultural messages regarding body size. Therefore, it is possible that the salience of macrolevel sociocultural messages regarding weight and shape could be amplified by partners who have deeply internalized these messages and reinforce them within the romantic relationship, or diminished by partners who have developed an effective defense against these messages and challenge their tenants to discount their importance within the romantic relationship.

Published literature has yet to apply an ecological systems framework to ED symptoms in sexual minorities; however, previous research indicates these models are a useful approach to examine the physical and mental health experiences of sexual minority individuals more generally [18–20]. Similar approaches such as the tripartite model, which emphasizes the role of social factors in relation to ED risk, have been supported among sexual minority samples [21–23]. Therefore, it appears that to better understand ED risk and protective factors for sexual minority individuals, more attention is needed toward sociocultural and relationship factors.

### Internalization of sociocultural attitudes

Some research supports the notion that internalization of sociocultural attitudes may be a particularly relevant factor in the development of EDs for sexual minority individuals. Much of the literature on this topic has examined this question separately for men and women.

For men, the relationships between media and body dissatisfaction and between media and lower self-esteem have been shown to be stronger for gay men compared to heterosexual men [24]. Some have theorized that social media use may relate to greater internalization of sociocultural attitudes that promote negative body image through processes such as social comparison, self-objectification, and self-surveillance, and that this in turn heightens ED risk for sexual minority men [25]. These ideas are supported by evidence indicating that social media use is linked to muscularity dissatisfaction and ED symptoms among sexual minority men [26]. Similarly, greater exposure to idealized muscular images through fitness magazines has been linked to body dissatisfaction, greater physique-related anxiety, and greater drives for both muscularity and thinness for gay men [27]. In addition to the exposure effects of images, some research has found that sexual minority men are more influenced than heterosexual men by images in advertisements focusing on physical appearance; this heightened sensitivity to internalization of social messages may explain the relationship between sexual orientation and ED symptoms for men [28]. These findings further indicate that gay men are more influenced by social messages through media than heterosexual men; greater influence by these messages then may mediate the effect of sexual orientation on ED symptoms [29].

For women, the negative effects of internalization of messages in the social environment pertaining to weight and eating have been well-documented [30, 31]; however, less is known about this among sexual minority women. Evidence synthesized in a systematic review indicates that many of the sociocultural risk factors related to negative body image and ED symptoms among heterosexual women extend to sexual minority women as well [32]. This research suggests that the internalization of sociocultural attitudes for sexual minority women may be similar to what has already been found for women in general. Some evidence consistent with these findings supports that internalization of beauty ideals predicts negative body image among sexual minority women [33]. More recent research also indicates that media-based appearance-related pressures and internalization of thin and muscular ideals are similar for lesbian, bisexual and heterosexual women, and are linked to comparable levels of ED symptoms across sexual orientation groups [21]. However, some research has found contrasting results. For instance, some have found that lesbian women have lower internalization of sociocultural beauty standards [34] and lower body dissatisfaction [35] compared to heterosexual women despite having similar or higher levels of ED symptoms. Others have found that lesbian women have lower appearance-related pressures from peers than heterosexual and bisexual women [21]. As such, greater attention needs to be paid to the ways in which internalization of sociocultural attitudes relate to ED symptoms in sexual minority women.

### Relationship factors

Relationship factors are also an important consideration when examining ED symptoms in sexual minority individuals. The literature is mixed however regarding the nature of the association between romantic relationship factors and ED symptoms. Several studies have indicated relationships may include potentially harmful effects for ED symptoms in sexual minority individuals. For instance, pressures from significant others to have an “ideal” body are strongly associated with ED symptoms for sexual minority men and women [36]. Additionally, having a partner who is of lower BMI can confer risk for ED symptoms for sexual minority men and women [37]. Within the context of romantic relationships, gay men’s preferences in partners have been found to be influenced by attractiveness and explained by internalization of the thin ideal and dissatisfaction related to weight and shape [15]. This finding suggests a high degree of importance is placed on attractiveness and physique in men’s same-gender relationships. Notably though, this was not found among lesbian women in the same study [15]. Gay men have been shown to be more likely to influence and monitor their partners’ eating habits compared to heterosexual men and compared to lesbian and heterosexual women [38]. Taken together, previous research suggests the importance of investigating gender differences in studies of relationship factors and ED symptoms among sexual minority individuals.

In addition to the studies that have pointed to a potentially negative role of romantic relationships, other work suggests being in a committed relationship may protect against risk for ED symptoms for sexual minority individuals. For instance, evidence suggests that gay men overestimate the degree to which other gay men are attracted to thin and muscular physiques; this leads to heightened body dissatisfaction for those who are single, but not for those in committed romantic relationships [39]. Similarly, for sexual minority men, being single has been linked to greater restrictive ED symptoms [40] and greater drive for thinness both cross-sectionally [41], and longitudinally over a 10-year period [42]. Gay men in stable relationships have been found to have lower ED symptoms compared to those who were not; however, no difference in ED symptoms has been found based on relationship status for lesbian women [43]. Although less is known about the effect of being in a relationship on ED-related risk factors for lesbian women, related research shows that being unpartnered is related to greater depression, anxiety, and alcohol use for this group [8, 44]. This suggest that being in a relationship confers mental health benefits for lesbian women that may extend to EDs.

However, relationship status alone is not the only factor for consideration, as not all relationships are equally protective. Lower relationship satisfaction has been correlated cross-sectionally with greater bulimic ED symptoms in sexual minority men [40]. Similarly, for sexual minority men, lower baseline satisfaction in their relationships was predictive of a trajectory toward a greater drive for thinness over a 10-year period [42]. Although no published research has examined the effect of relationship quality on ED symptoms among lesbian women, related work demonstrates that relationship satisfaction is a significant contributor to depression risk [45], and it is possible this may hold for EDs as well. This finding suggests that factors found within satisfying committed relationships, such as stability, support, and compassion, may underlie the benefits that being partnered may carry. That is, the quality of the relationship, rather than simply one’s relationship status, may be instrumental in understanding the relationships in association to ED symptoms.

To our knowledge, no previous research exists to examine whether a positive relationship can protect against the damaging effects of internalizing negative societal messages about shape and weight. It is possible that the broader attitudes that are held by Western cultures that emphasize a lean build may carry less importance when one’s body is loved and appreciated by a partner in a satisfying and committed relationship. Similarly, the support and validation of worth that one receives from a partner may protect against negative sociocultural messages on eating behavior.

### Current study

In sum, sociocultural and relationship factors warrant additional consideration as social environmental factors in relation to ED symptoms among sexual minority individuals. Few studies have considered the ways in which both overarching sociocultural influences and close romantic relationships affect ED symptoms in sexual minority individuals despite the elevated prevalence of ED concerns in this group. There is a demonstrated need to better understand risk and protective factors for ED symptoms among sexual minority individuals. In particular, very little work exists examining sociocultural and relationship factors within the context of same-gender couples, or across sexual minority men and women. There is a precedent in the literature of examining ED symptoms separately for sexual minority men and women and drawing inferences about risk; however, it is important to determine whether gender differences exist by comparing men and women directly.

The current study plans to fill these gaps by examining the relation of internalization of sociocultural attitudes and relationship quality to ED symptoms among sexual minority individuals in relationships with same-gender partners. The ability to examine these questions in a sample composed of couples allows us to control for relationship status and focus more on the quality of the relationship. This will allow us to determine whether the associations that may be observed are consistent across same-gender relationships, or whether differences between men and women may be present.

Our aims are as follows:

1. We aim to evaluate gender differences in the internalization of sociocultural attitudes, relationship quality, and ED symptoms.
2. We aim to examine the association of internalization of sociocultural attitudes and relationship quality to ED symptoms.
3. We aim to evaluate the interaction of internalization of sociocultural attitudes and relationship quality on ED symptoms. Specifically, we seek to learn whether the effect of a high-quality romantic relationship can buffer against the internalization of sociocultural attitudes pertaining to weight and shape, thus protecting against ED symptoms.
4. We plan to explore the conditional association of gender by examining:
  - The interaction of internalization of sociocultural attitudes and gender on ED symptoms.
  - The interaction of relationship quality and gender on ED symptoms.

## Method

### Participants

The sample for this study was derived from a larger study focused on romantic relationships and health, and was comprised of 144 men in same-gender relationships (72 couples) and 144 women in same-gender relationships (72 couples). Participant characteristics by gender are reported in Table 1.

**Table 1:** Descriptive statistics for participant characteristics and key variables by gender

	Men (*n* = 144)		Women (*n* = 144)		Gender differences	
	Range	% or M(SD)	Range	% or M(SD)		
*Within-couple variables*					*OR (95% CI)*	
Race/ethnicity, %	–		–		0.99 (0.51, 1.94)^a^	
European American/White		70.8%		70.8%		
African American/Black		11.1%		17.4%		
Hispanic/Latinx		11.8%		4.9%		
Asian American		2.1%		2.8%		
Other		4.2%		4.2%		
Education, %	–	57.6%	–	59.7%	1.09 (0.62, 1.93)^b^	
Less than high school degree		1.4%		2.8%		
High school degree		3.5%		6.3%		
Some college or vocational training		37.5%		31.2%		
Bachelor’s degree		20.1%		26.4%		
More than a bachelor’s degree		37.5%		33.3%		
					*B (SE)*	*pr*
Age	19–71	34.13 (12.31)	18–65	33.32 (10.21)	− 0.14 (1.78)	− 0.01
BMI	16.89–40.92	26.28 (4.59)	16.88–61.83	29.38 (8.23)	3.10 (0.91)***	**0.28**
ISA	1–5	2.81 (1.02)	1–4.22	2.06 (0.84)	− 0.75 (0.12)***	− **0.45**
Relationship quality	69–133	107.82 (11.06)	56–133	108.80 (12.15)	0.99 (1.60)	0.05
Eating attitudes	0–46	8.42 (7.69)	0–35	6.93 (6.75)	− 1.49 (0.87)	− 0.14
Cognitive restraint	0–100	41.635 (22.20)	0–84.13	43.25 (18.85)	1.62 (2.56)	0.05
Emotional eating	0–100	41.90 (31.45)	0–100	37.81 (26.71)	− 4.09 (3.49)	− 0.10
Uncontrolled eating	0–96.30	39.68 (23.11)	0–96.30	34.47 (18.74)	− 5.22 (2.51)*	− **0.17**
*Between-couples variables*						
Relationship length, years	0.50–61.50	6.41 (9.94)	0.50–19.00	4.69 (4.47)	*t*(142) = 1.36	*d* = 0.23
Cohabitation, % yes	–	83.1%	–	84.7%	*χ*^2^ (1, *N* = 143) = 0.07	

Values that appear in bold reach the p < .05 threshold for statistical significance

Men = 0; women = 1. For within-couple variables, gender differences were tested by examining gender as a predictor in multilevel models; for between-couples variables, independent samples t-tests (relationship length) or chi-square tests (cohabitation) were conducted

*ISA* internalization of sociocultural attitudes toward appearance, *pr* partial correlation

*p < 0.05. **p < 0.01. ***p < 0.001

^a^Test of the difference between white versus non-white

^b^Test of the difference between less than a bachelor’s degree versus bachelor’s degree or more

### Procedure

Participants were recruited from the greater Philadelphia, PA area through local health and LGBTQIA+ advocacy groups and events, as well as through advertisements in print and online periodicals. Two advertisements were circulated, one recruiting for “men in relationships with men” and one recruiting for “women in relationships with women.” Eligibility criteria included being 18 years of age or older, and in a committed same-gender relationship for a minimum of 6 months; exclusion criteria included being diagnosed with a chronic or dietary-related health condition (e.g., diabetes). Eligible couples came to the researcher’s lab together and completed surveys (in separate rooms) and participated in other study-related tasks focused on romantic relationships and health. We recruited couples as dyads because we were interested in having relationship quality indicators from both individuals in the couples represented in the dataset. Before participating, all individuals provided informed consent. Each individual was compensated with $50 ($100 per couple). This study was approved by the Institutional Review Board at the university where the data were collected.

### Measures

#### Independent variables

##### Internalization of sociocultural attitudes toward appearance

To assess sociocultural attitudes toward appearance, we used 9 items from the internalization subscale of The Sociocultural Attitudes Towards Appearance Scale-3 (SATAQ-3). Participants indicated on a 5-point scale (1 = “definitely disagree,” 5 = “definitely agree”) the extent to which they agreed with statements about sociocultural attitudes toward appearance (e.g., “TV programs are an important source of information about fashion and ‘being attractive’”). This measure demonstrates excellent convergent validity and internal consistency [46]. It has been used widely in sexual minority samples [6, 15, 28, 34, 47]. Items were averaged to create a composite scale (α = 0.91).

##### Relationship quality

To assess relationship quality, we used 15 items from the Marital Interaction Scale [48]. Two of the four subscales were used in this study, which measured love (ten items) and conflict (five items; reverse coded). References to spouses were changed to “partner” or “significant other” following procedures used in previous research with this measure [49]. Participants responded on a 9-point scale (1 = “not at all,” 9 = “very much*”*) the extent to which they viewed their partner and relationship (e.g., love item, “How close do you feel toward your partner?”; conflict item, “How often do you and your partner argue with one another?”). Items were summed to create a composite scale (α = 0.84). This measure has been used previously in samples including lesbian and gay couples [49].

#### Dependent variables

##### Disordered eating

To assess disordered eating, we used two different measures. First, the 26-item eating attitudes test (EAT-26) was used. This questionnaire is used clinically, and in research settings, to screen for EDs symptoms. Test–retest and internal consistency reliability is high, and validity has been established with regard to clinical indicators [50]. Participants responded on a 6-point scale (0 = “never,” 5 = “always”) the extent to which they agreed with statements about eating behaviors, attitudes toward food, and weight concerns (e.g., “I avoid eating when I am hungry”). Using standard scoring procedures, 25 items were recoded so that 0 = “never,” “rarely,” or “sometimes;” 1 = “often;” 2 = “usually;” and 3 = “always” (one item, “enjoy trying rich new foods” was recoded so that 0 = “always,” “usually,” or “often;” 1 = “sometimes;” 2 = “rarely;” and 3 = “never”). Items were then summed to create a composite measure (α = 0.80). This measure has been used previously in samples including sexual minority individuals [34, 51, 52]. Next, the 18-item Three-Factor Eating Questionnaire (TFEQ-18) was used to assess three subscales of disordered eating. This measure has demonstrated convergent and discriminant validity [53, 54]. The subscales are as follows: (1) cognitive restraint (6 items; e.g., “I deliberately take small helpings as a means of controlling my weight;” α = 0.78); (2) emotional eating (3 items; e.g., “When I feel anxious, I find myself eating;” α = 0.88); and (3) uncontrolled eating (9 items; e.g., “Sometimes when I start eating, I just can't seem to stop;” α = 0.88). As items were on different response scales, items were first standardized to a 0–100 scale and then averaged to create composites.

#### Descriptive variables

Participants completed the Klein Sexual Orientation Grid to provide descriptive information relating to their sexual orientation. This questionnaire has demonstrated reliability and validity in previous research [55]. Specifically, we utilized the self-identification item which asks respondents to indicate how they currently identify their sexual orientation on a scale ranging from 0 (heterosexual only) to 6 (gay/lesbian only). This item has been shown to be the best predictor of the overall response pattern for this questionnaire [55]. We considered covariates using a two-pronged approach that involved conceptual and statistical criteria. First, we considered covariates that have been reported in the literature to be relevant to disordered eating and sexual minority individuals, including gender, age, body mass index (BMI), and outness. Because we specifically sought “men in relationships with men” and “women in relationships with women” we inferred reasonably strong identification with these respective gender identities within the couple and took participants endorsement of their same-gender relationship at face value and represented it as such within our data rather than assessing gender or biological sex directly. Outness was assessed as the sum of 11 items from the Outness Inventory [56]. Second, we considered variables that had significant bivariate associations with at least one of the disordered eating measures that were included in analyses; only gender and BMI met this criterion and were thus included in analyses. Gender also was examined as a between-couple moderator in models that tested Aim 4 (0 = man, 1 = woman).

### Analytic plan

SPSS version 28 and HLM 7 were used for descriptive analyses and to test study aims. Prior to analyses, data were checked for completeness; the only variable on which data were missing was eating attitudes (*n* = 1). Given the very small amount of missing data, listwise deletion was used. Although the focus of our analyses is on the association between individual-level variables (level 1), all analyses used multilevel models, given the dependent, nested nature of the data. The multilevel models were analyzed using full maximum likelihood; because dyadic analyses limit the number of random effects parameters estimated, random slopes were not estimated [57]. We did not estimate random slopes because according to p. 2 of McMahon and colleagues "dyadic multilevel analysis incorporating both random intercepts and slopes will result in an overdetermined model—one with too many parameters to be estimated given the number of covariance elements available" [58].

To test Aim 1, gender differences were examined in key variables by including gender as an independent variable in multilevel models. To test Aim 2, four models were run to test the association of internalization of sociocultural attitudes toward appearance and relationship quality with each measure of disordered eating; both independent variables were included in the same model to determine the unique association of each predictor, after controlling for the other independent variable. To test Aim 3, four models were run to examine relationship quality as a moderator of the association between internalization of sociocultural attitudes toward appearance and each measure of disordered eating; independent variables were grand-mean centered prior to calculating interaction terms. Finally, four additional models were run to examine gender as a cross-level moderator of internalization of sociocultural attitudes toward appearance (Aim 4a) and relationship quality (Aim 4b) for each measure of disordered eating. All models controlled for BMI. The nature of significant interactions with gender were determined by calculating simple slopes for each gender and at ± 1*SD* of the mean for the independent variables. The *t* statistics from the multilevel models were transformed into partial correlations to provide a measure of individual effect size.

## Results

### Descriptive statistics

Men in our sample rated their sexual identity in the following ways: mostly heterosexual: 0.7%; equally heterosexual and gay: 2.1%; gay somewhat more: 3.5%; gay mostly: 13.3%; and gay only: 80.4%. Women in our sample rated their sexual identity in the following ways: heterosexual somewhat more: 0.7%; equally heterosexual and lesbian: 7.0%; lesbian somewhat more: 7.0%; lesbian mostly: 23.9%; and lesbian only: 61.3%.

Table 1 presents means and standard deviations for participant characteristics and key variables by gender. As shown, women had significantly higher BMIs than men. Two additional significant gender differences emerged in testing Aim 1: compared to women, men reported greater (1) internalization of sociocultural attitudes toward appearance and (2) uncontrolled eating. To determine concordance among partners in key variables, we examined pairwise intraclass correlations [59]. Results revealed that internalization of sociocultural attitudes toward appearance (ISA) and relationship quality were somewhat concordant among partners, reflected by a small to moderate correlation (r_ISA_: = 0.35, *p* < 0.001; r_relationship quality_ = 0.38, *p* < 0.001). Among eating disorder variables, only cognitive restraint had a significant, albeit small correlation, among partners (r_cognitive restraint_ = 0.12, *p* = 0.04). There was no significant association among partners for the other eating disorder variables (r_eating attitudes_ = 0.05, *p* = 0.41; r_emotional eating_ = 0.04, *p* = 45; r_uncontrollled eating_ = 0.05, *p* = 0.40).

### Study aims

Table 2 presents results from multilevel models that tested Aims 2–4. Results revealed that internalization of sociocultural attitudes toward appearance was positively and significantly related to all measures of disordered eating; specifically, the more individuals reported internalizing these attitudes, the greater disordered eating they also reported. Relationship quality was only significantly and negatively related to one measure of disordered eating—uncontrolled eating, although gender moderated this association. Specifically, for men, greater relationship quality was related to less uncontrolled eating (simple slope = − 0.74(0.15), *t* = − 5.03, *p* < 0.001), whereas this association was not significant for women (simple slope = − 0.10(0.14), *t* = − 0.72, *p* = 0.47). None of the other interactions were significant.

**Table 2:** Multilevel models examining sociocultural and relationship factors predicting disordered eating among sexual minority men and women

	Eating attitudes		Cognitive restraint		Emotional eating		Uncontrolled eating	
	*B *(*SE*)	*pr*	*B *(*SE*)	*pr*	*B *(*SE*)	*pr*	*B *(*SE*)	*pr*
*Main effects ****(Aim 2)***								
ISA	2.05 (0.44)***	**0.36**	5.82 (1.30)***	**0.35**	5.00 (1.79)**	**0.23**	3.27 (1.28)*	**0.21**
Relationship quality	− 0.04 (0.04)	− 0.09	0.11 (0.11)	0.09	− 0.25 (0.14)	− 0.15	− 0.39 (0.10)***	− **0.30**
Gender	− 0.68 (0.93)	− 0.06	5.10 (2.85)	0.15	− 3.42 (3.65)	− 0.08	− 3.89 (2.59)	− 0.13
BMI	0.25 (0.06)***	**0.32**	0.25 (0.18)	0.12	1.07 (0.25)***	**0.34**	0.48 (0.18)**	**0.22**
*Interaction effects*								
ISA * Relationship quality (**Aim 3**)	0.01 (0.03)	0.02	− 0.09 (0.09)	− 0.08	− 0.13 (0.13)	− 0.08	− 0.10 (0.09)	− 0.09
ISA * Gender (**Aim 4a**)	0.90 (0.91)	0.08	2.22 (2.67)	0.07	3.42 (3.67)	0.06	− 1.55 (2.60)	− 0.05
Relationship quality * Gender (**Aim 4b**)	− 0.07 (0.07)	− 0.08	0.20 (0.21)	0.08	0.56 (0.29)	0.16	0.64 (0.20)**	**0.26**

Values that appear in bold reach the p < .05 threshold for statistical significance

All models reported controlled for BMI. Models with interaction terms also included main effects, which are not reported here. Models were run separately for each outcome within each aim (Aims 4a and 4b were run the in same model)

*ISA* internalization of sociocultural attitudes toward appearance, *SE* standard error, *pr* partial correlation

*p < 0.05. **p < 0.01. ***p < 0.001

## Discussion

The goal of this study was to examine social environmental predictors of ED symptoms among men and women in same-gender relationships. Utilizing Bronfenbrenner’s [9] ecological systems theory as a framework, we considered the role of a microsystem factor (e.g., romantic relationship quality), and macrosystem factor (e.g., internalization of sociocultural attitudes toward appearance) in ED symptoms. In support of this model, both factors contributed to ED symptoms, but in different ways. Individuals who internalized sociocultural attitudes toward appearance to a greater extent reported more ED symptoms. Our moderation analysis indicated relationship quality was significantly related to uncontrolled eating, but only for men. That is, men who had higher quality relationships with their partners reported less uncontrolled eating.

Findings from our first study aim, examining gender differences in key study constructs, showed that men reported significantly higher internalization of sociocultural attitudes toward appearance and more uncontrolled eating. This finding indicates that the sexual minority women in our study were less sensitive to appearance pressures; however, as all of the women in our study were currently in relationships with other women, it is difficulty to parse whether this finding holds true for both lesbian women and those who may be interested in both same and other-gender partners. That is, because research demonstrates that lesbian women experience fewer appearance-related pressures than bisexual women [21], it is possible differences may exist within the sexual minority women in our sample. Similarly, greater sense of belonging within the lesbian community has previously demonstrated a protective effect [8]; those who do not identify as lesbian or feel strong affiliation with the LGBTQIA+ community may be more vulnerable to internalization of sociocultural attitudes. Additionally, personal gender conceptualization, past and potential future partners’ gender, and experiences of stigma, may be factors of relevance for internalization of sociocultural norms.

These findings also suggest that sexual minority men are more sensitive to sociocultural appearance pressures than sexual minority women. Sociocultural norms within gay communities promote the attractiveness of a lean, muscular body type [5, 6]. Significant yet small gender differences in BMI (i.e., lower BMI in sexual minority men as compared to women) may reflect this appearance pressure; perhaps men are modifying their eating and physical activities to achieve a fit and lean body. Men’s greater uncontrolled eating may also reflect distress related to these appearance pressures. For instance, they may actively restrict food to achieve a lean body, and then eat uncontrollably afterward from hunger. Uncontrolled eating may also be considered more socially acceptable for men than for women. Eating large quantities of food may be seen as representing men’s hearty appetite or a way for them to “bulk up” [60]. Binge eating disorder is more common than anorexia nervosa among men [61], suggesting that uncontrolled eating may be a more common eating concern for men than restriction.

Our second study aim was to examine the associations of internalization of sociocultural attitudes toward appearance and relationship quality with ED symptoms. There were significant associations for internalization of sociocultural attitudes toward appearance, but not relationship quality. Specifically, individuals who reported greater internalization of sociocultural attitudes toward appearance had more ED symptoms. Individuals who feel greater pressure to achieve sociocultural standards of thinness, which are narrow and therefore hard for many to achieve, may have more weight concerns and engage in more food restriction to limit weight gain. Previous research shows that gay men are more influenced by media messages about appearance than heterosexual men, which may relate to higher ED symptoms [28, 29]. Our findings support prior work by demonstrating that the extent to which sexual minority men internalize sociocultural attitudes toward appearance seems to relate to a relatively higher level of ED symptoms. Appearance-related pressures seemed to relate to ED symptoms for sexual minority women in our sample as well, consistent with prior research [21, 32]. Thus, sexual minority men and women are susceptible to sociocultural pressures (although men more so than women) and these pressures relate to their eating behavior in similar ways.

Our third and fourth study aims focused on examining interactions between internalization of sociocultural attitudes and relationship quality on ED symptoms, including the conditional association of gender. Relationship quality was significantly associated with uncontrolled eating, although gender moderated this association. Specifically, men who had higher quality romantic relationships reported significantly less uncontrolled eating; this association was not significant for women. Having a higher quality romantic relationship seemed to serve as a buffer for some of men’s ED symptoms. Given that gay men’s lived experiences exist within a context where there is pressure to have an athletic build [5, 6], it is important to identify factors that can offer protection from this pressure. Partners may provide positive feedback about appearance, and create a context for adaptive eating habits (e.g., preparing and eating meals together). This kind of support and encouragement may reduce appearance-related pressures and, in turn, ED symptoms. These effects may be particularly salient for the men in our study. Social norms encourage women to be more relationship-focused than men [62], so men who willingly participate in a relationship study may be more relational and particularly open to influence from their partners. Given the dearth of literature on relationship factors and disordered eating, it is unclear why, but further research is needed to better understand why relationship quality was not protective for women or for other types of disordered eating for gay men.

This study has several limitations. The sample was composed of mostly White individuals with at least a bachelor’s degree from the northeast U.S., limiting the generalizability of findings. Examination of more diverse samples in terms of race, education, and geographic region, is needed. We excluded individuals with chronic or dietary health conditions in the interest of ensuring that differences in eating behaviors and BMI were not reflective of medical diagnoses; however, we recognize that chronic health conditions are common and by excluding this subset of the population our generalizability may be reduced. Further, participants self-identified as being in a same-gender relationship, but we did not include a direct, individual assessment of gender identity or expression, so we cannot definitively state whether these data are inclusive of transgender individuals. Future studies should actively recruit transgender individuals and assess gender identity in their examination of these research topics, as ED prevalence appears to be high among transgender individuals [63]. Additionally, including examples of tailored media, such as social media, may be useful in future examinations of Ecological Systems Theory. We also recognize that measures (e.g., Dual-energy X-ray absorptiometry) that can directly assess lean mass versus adiposity might yield greater specificity regarding the role of BMI in our sample. We included BMI as a control variable in our models examining gender differences, although BMI could be modeled differently (e.g., as an explanatory variable between predictors and eating behaviors). Although we could not explore this question with our analyses, future research using longitudinal methods could examine this idea. In addition, the cross-sectional nature of the study precludes a determination of the direction of effects; it is possible that individuals who already have disordered eating may be more likely to be sensitive to sociocultural influences. Finally, we examined only two components of Bronfenbrenner’s [9] ecological systems model (microsystem and macrosystem). The mesosystem and exosystem may warrant further study in future work on contextual influences on ED symptoms.

## Conclusion

This study adds to the literature on disordered eating among sexual minority men and women, who are at high risk for ED symptoms [2]. Men in this sample internalized sociocultural attitudes toward appearance to a greater extent than women, although both men and women who reported greater internalization of these norms had higher ED symptoms. For men (but not women), relationship quality seems to act as a buffer against certain ED symptoms. Having a supportive partner who can provide positive feedback about appearance and support adaptive eating behaviors may be important for mitigating some types of ED symptoms for sexual minority men. Findings suggest that both micro (e.g., relationship quality) and macro (e.g., sociocultural attitudes toward appearance) factors in the social environment are important in understanding ED symptoms in sexual minority men and women. Clinicians treating ED symptoms among sexual minority populations may want to consider the role of romantic relationships and sociocultural appearance pressures in the presentation of weight and eating concerns. Greater incorporation of partners as supports in therapy may be particularly beneficial in addressing ED concerns among sexual minority men. Better understanding of the ways in which evidence-based ED prevention programs are effective in protecting against the impact of negative sociocultural messages for sexual minority individuals is necessary.

## Data Availability

The data that support the findings of this study are available on request from the corresponding author. The data are not publicly available due to privacy or ethical restrictions.

## Abbreviations

ED: Eating disorder
BMI: Body mass index
ISA: Internalization of sociocultural attitudes toward appearance
LGBTQIA+: Lesbian, gay, bisexual, transgender, queer, intersex, asexual and others who are part of the community
